# Single-cell sequencing reveals MYC targeting gene MAD2L1 is associated with prostate cancer bone metastasis tumor dormancy

**DOI:** 10.1186/s12894-022-00991-z

**Published:** 2022-03-19

**Authors:** Xing Wang, Jiandi Yu, Junfeng Yan, Kun Peng, Haiyong Zhou

**Affiliations:** Department of Urology, Zhejiang Hospital, #1229, Gudun Road, Hangzhou, 310030 China

**Keywords:** Single cell sequencing, Bioinformatics, Prostate cancer, Biomarkers, MAD2L1 gene

## Abstract

**Background:**

Among malignant tumors, bone metastasis is frequently associated with prostate cancer which is seen in about 80% of patients. During cancer treatments, some tumor cells switch to a "dormant mode" to help tumor cells avoid attack from the immune system and anti-tumor therapies. In this dormant mode, tumor cells can be resuscitated, causing cancer to reoccur. The generally accepted explanation for this phenomenon is that the tumor cells have spread to the bone marrow before treatment and are dormant in the bone marrow. However, the key mechanism for inducing and maintaining the dormancy of these prostate cancer disseminated tumor cells in the bone marrow is still unclear. Therefore, studying the dormancy mechanism of tumor cells in bone metastasis is of great significance for the treatment and the prevention of recurrence of prostate cancer.

**Methods:**

We obtained single-cell RNA-seq data of tumors from mouse models of prostate cancer bone metastasis mouse model numbered (GSE147150) from the GEO database, and obtained RNA-seq expression data and clinical information from The Cancer Genome Atlas Program (TCGA) of prostate cancer patients from the USCS Xena database. Screening of differential genes and annotation of GO functions were performed separately. Subsequently, the screened differential genes were compared and analyzed with 50 classic Hallmark signaling pathways, and the prognosis analysis of prostate cancer patients in TCGA data was performed to discover the key genes of the dormant mechanism of tumor cells in bone metastasis, and obtain new biomarkers that can be used to predict the prognosis of patients.

**Results:**

A total of 378 differentially expressed genes were screened, of which 293 were significantly up-regulated and 85 were significantly down-regulated. Among them, the up-regulated genes were mainly related to the immune response, and the down-regulated genes were mainly related to the cell cycle. Through GSVA (Gene set variation analysis), it is found that there are differences in a total of 3 signal pathways: COMPLEMENT, MYC_TARGETS_V1 and MYC_TARGETS_V2. By comparing and analyzing the significantly down-regulated genes in dormant tumor cells with MYC_TARGETS_V1, MYC_TARGETS_V2, three significantly down-regulated genes were obtained: Ccna2, Mad2L1 and Plk1.

**Conclusion:**

In summary, our findings indicate that the MYC targeting gene Mad2L1 is potentially related to the dormancy mechanism of prostate cancer. At the same time, Mad2L1, a gene associated with dormant prostate cancer cells, may be used as a biomarker for prognostic survival.

## Background

Prostate cancer (PCa) is a malignant tumor of the urinary system that often occurs in middle-aged and elderly men. It is the second leading cause of death from tumors in men worldwide. Nearly 350,000 patients die of PCa each year [1, 2]. Because PCa has no characteristic clinical symptoms in the early stage, it is often in the middle or late stage when it is discovered, and about 80% of advanced PCa patients will be accompanied by bone metastasis. Once bone metastasis occurs, the patient will have a reduced likelihood of recovery [3–5]. Bone metastasis of PCa can cause a series of complications, such as pain, pathological fractures, and paraplegia, which severely reduce the quality of life of patient. In addition, after bone metastasis occurs, the prognosis is generally poor, and the survival period is also significantly shortened [6, 7].

During cancer treatments, some tumor cells switch to a "dormant mode" to help tumor cells avoid attack from the immune system and anti-tumor therapies. In this dormant mode, tumor cells can be resuscitated, causing cancer to reoccur [8–11]. Tumor dormancy is one of the biological characteristics of malignant tumors. Tumor dormancy is also an important reason why tumors are clinically difficult to cure, due to metastasis and recurrence [12–14]. Tumor dormancy mechanisms are diverse and complex, as they involve a series of related genes, cytokines and proteins, etc. [15, 16]. Changes in the tumor microenvironment will cause dormant tumor cells to re-proliferate, leading to cancer recurrence. Tumor dormancy is very common clinically among many malignant tumors. Many patients will relapse for a long period of time after intense treatment of the primary tumor. The study of the dormant mechanism of tumor cells in bone metastasis is therefore, of great significance for the treatment of PCa patients [17].

This study uses the GEO database and USCS Xena database to reveal for the first time that the MYC targeting gene MAD2L1 is potentially related to tumor dormancy mechanisms. The high expression of MYC target gene MAD2L1 in PCa patients will lead to a significantly poorer prognosis. This study provides new research ideas for the dormancy mechanism of PCa bone metastases. At the same time, it provides a new prognostic target for PCa.

## Methods

### Data sources

We accessed the GSE147150 data set from the GEO database (https://www.ncbi.nlm.nih.gov/geo/) to obtain the single cell RNA-seq data of the RM1 bone metastasis cells of mouse PCa. Prostate cancer cell-intrinsic interferon signaling regulates dormancy and metastatic outgrowth in bone [18]. The Seurat package was used to process scRNA-seq data, and perform quality control on cells and genes. The screening criteria were that each cell must express at least 200 genes, and each gene is expressed in at least 3 cells. Through the standardized single-cell analysis process, we carried out standardization, principal component analysis and K-Nearest Neighbor clustering on the data, and used the UMAP algorithm for data visualization analysis. We accessed the RNA-seq expression data and clinical information of PCa patients in the TCGA dataset from the USCS Xena database (http://tcga.xenahubs.net).

### Differentially expressed gene screening and functional annotation

In order to screen the differentially expressed genes in dormant tumor cells and proliferating tumor cells, we first extracted 2,000 Variable genes using the "FindVariableFeatures" function in the Seurat package. Afterwards, the Wilcoxon rank sum test was used to further screen the differentially expressed genes between the two groups of cells (*P* < 0.05). Subsequently, we also performed functional annotations on the differential genes selected, and combined with the DAVID tool (https://david-d.ncifcrf.gov/) to interpret the biological functions of the differentially expressed genes from the two aspects of GO terms and KEGG pathway.

### Characteristics of dormant tumor cell signaling pathway

We have collected 50 classic Hallmark signal path data from the MsigDB database (http://software.broadinstitute.org/gsea/msigdb).Molecular signatures database (MSigDB) 3.0 [19], Gene set enrichment analysis: A knowledge-based approach for interpreting genome-wide expression profiles [20]. Using the GSVA algorithm, we scored each tumor single cell, and converted the original single cell gene expression matrix into a single cell GSVA score matrix of 50 Hallmark signaling pathways. Combined with the student’s *t* test, screening of signal pathways in which there was a significant difference between dormant tumor cells and proliferating tumor cells (*p* < 0.05).

### Patient survival analysis

Combining the clinical information of PCa patients in The Cancer Genome Atlas (TCGA) to evaluate the impact of genes (By analyzing the mouse single-cell data set, the tumor cell dormancy-related gene was found, and then the TCGA PRAD human tissue sequencing data was used for survival analysis to verify the value of the gene, and the homologous conversion of the mouse gene was performed.) related to dormant characteristics in metastatic tumor cells on the prognosis of PCa patients. We divided patients into high-expression and low-expression groups based on gene expression values, and constructed a Cox proportional hazard regression model. The log-rank test was used to evaluate the efficacy of feature genes in predicting the prognosis of patients, and the screening standard was a *P*-value < 0.05 by log-rank test.

## Results

### Dormant tumor cell characteristics

**Fig. 1:**
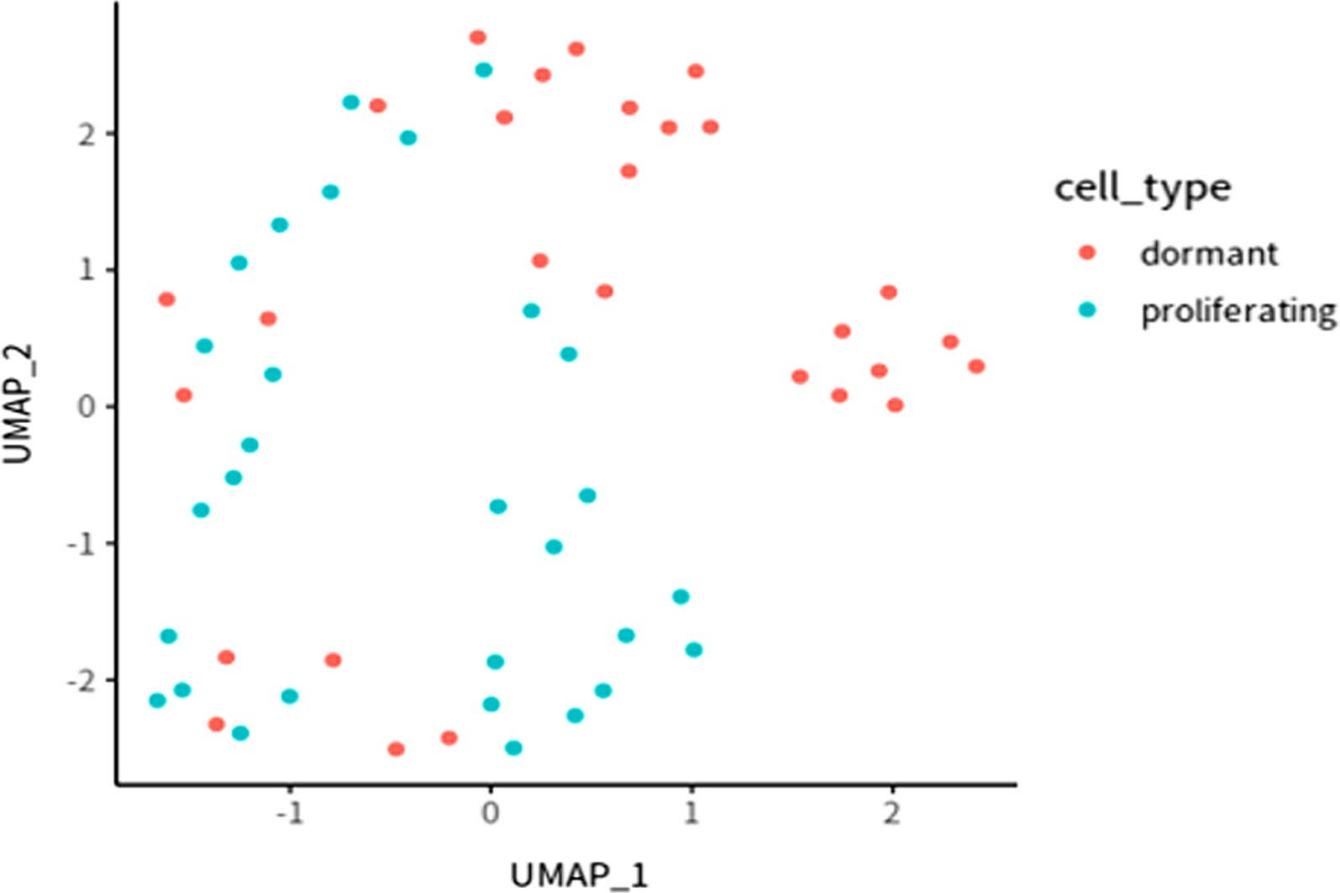
Dormant tumor cell characteristics

We obtained tumor single cell RNA-seq data collected from the RM1 bone metastasis mouse model from the GSE147150 data set [18], which contained 13,005 gene expression profiles of 57 tumor cells. In the end, we obtained a 12,468 gene expression profile containing 28 dormant tumor cells and 29 proliferating tumor cells which were classified by GSE147150 (Fig. 1). After data standardization and PCa dimensionality reduction, we used the UMAP algorithm to visually analyze the expression data, where the min.dist parameter was set to 0.1.

### Analysis of differential genes in dormant tumor cells

**Fig. 2:**
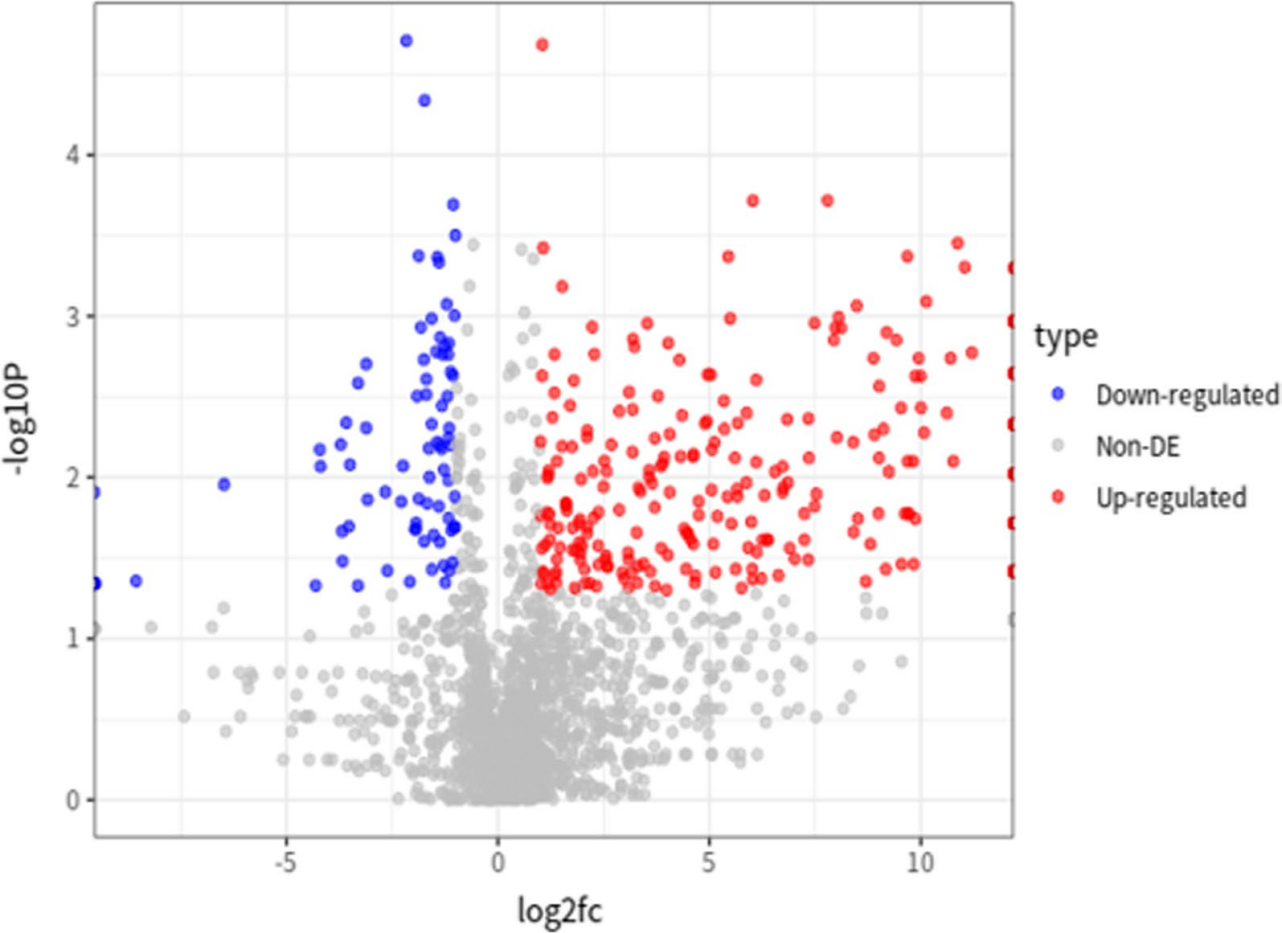
Differential gene volcano plot of dormant tumor cells

**Fig. 3:**
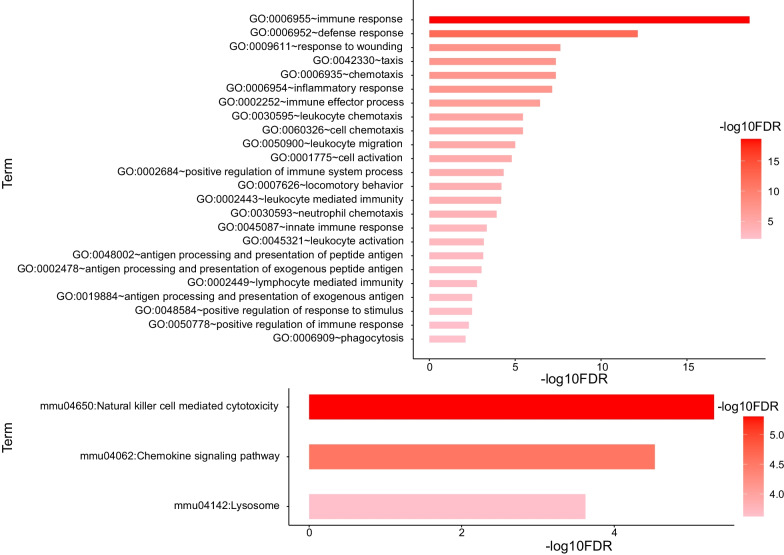
Up-regulated gene ontology (GO) annotation

**Fig. 4:**
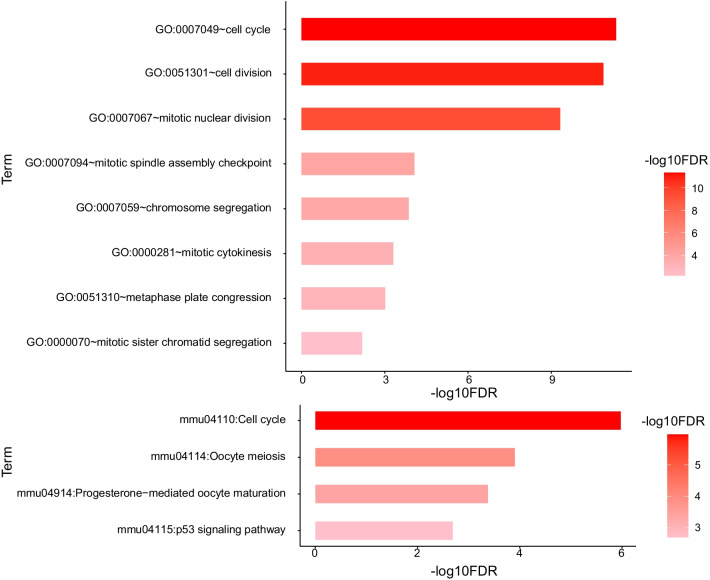
Down-regulated gene ontology (GO) annotation

According to the visualization results, we found that dormant tumor cells have different expression characteristics from ordinary tumor cells that are undergoing proliferation. Therefore, we conducted a screening of differentially expressed genes to obtain the characteristics of genes specifically expressed in dormant tumors to explain the possible dormant mechanism of tumors. First, use the "FindVariableFeatures" function in the Seurat package to extract 2000 highly variable genes, then combined with the Wilcoxon rank sum test, 85 significantly down-regulated genes and 293 significantly up-regulated genes were further screened (Fig. 2). Among them, the up-regulated genes are closely related to the immune response (Fig. 3), and the down-regulated genes are all related to the cell cycle (Fig. 4), which is also in line with the feature that dormant cells no longer have a strong proliferation phenomenon.

### Functional characteristics of dormant tumor cells

**Fig. 5:**
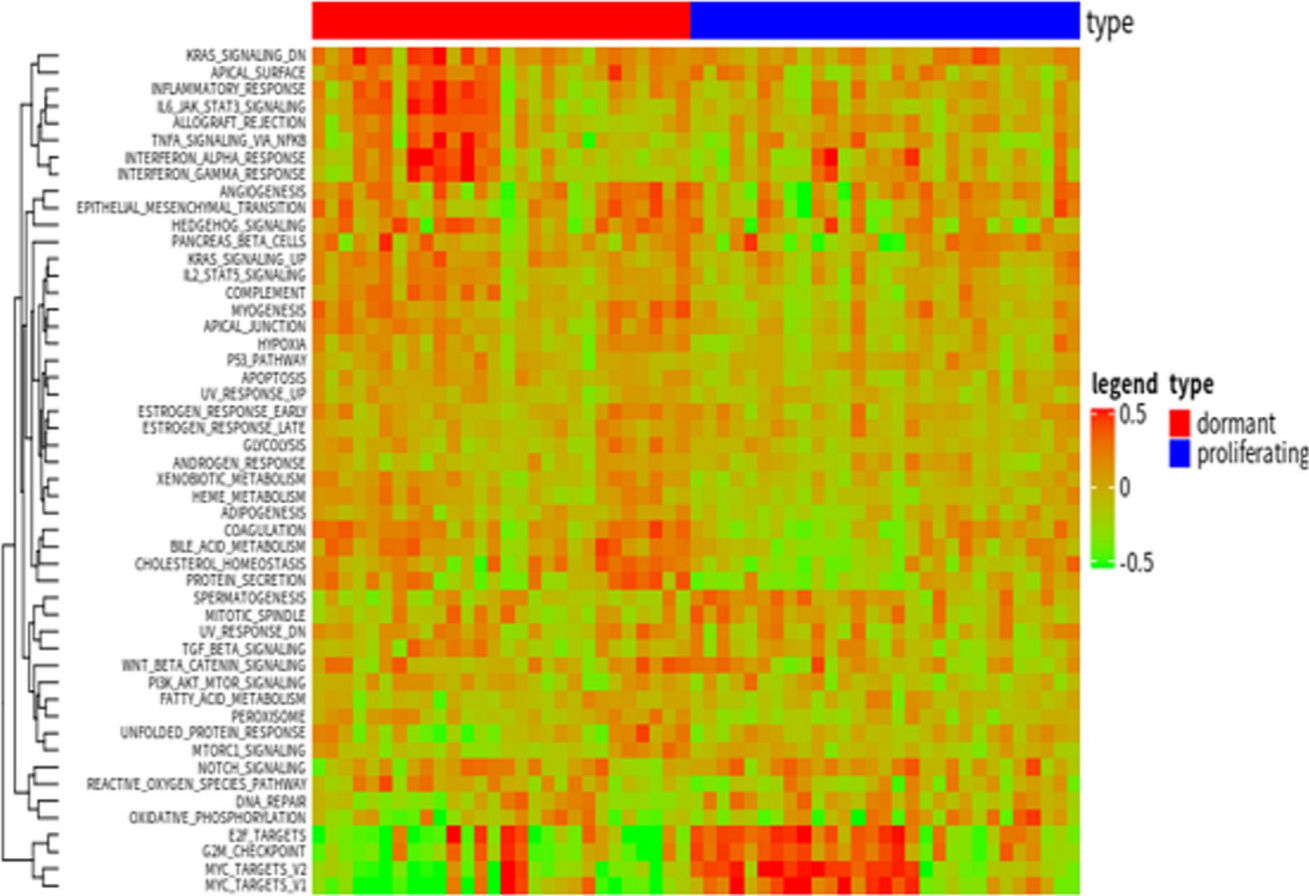
Diagram of gene expression difference between proliferating tumor cells and dormant tumor cells

**Fig. 6:**
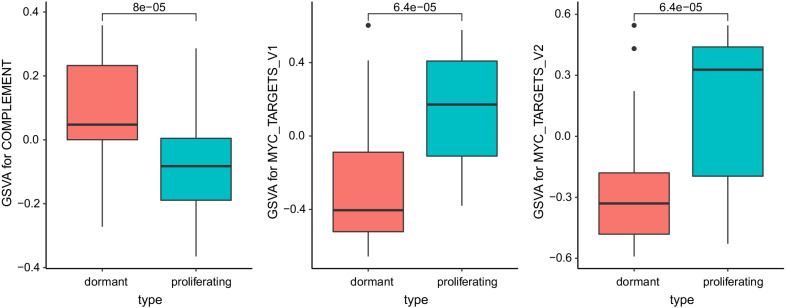
Signal pathway differences between dormant tumor cells and normal proliferating tumor cells

**Fig. 7:**
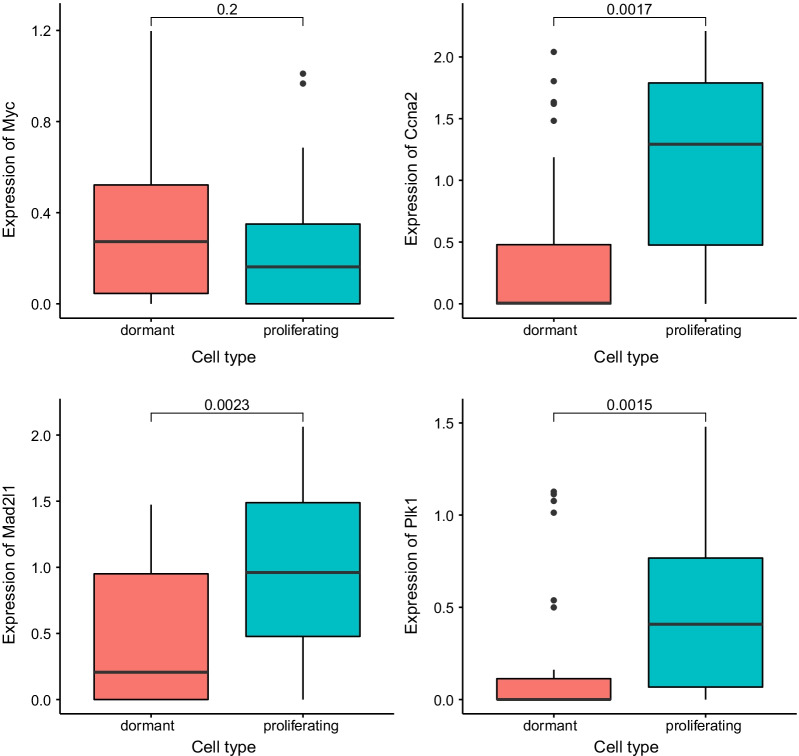
Signal pathway differences between dormant tumor cells and normal proliferating tumor cells

In order to further study the dormancy mechanism of tumor cells, we obtained 50 classic hallmark signaling pathway data from MsigDB. Starting from these 50 signaling pathways, we explored the functional differences between dormant tumor cells and normal proliferating tumor cells (Fig. 5). First, we used the biomaRt package to obtain the homologous genes of humans and mice, and converted the human genes in MsigDB into the corresponding mouse homologous genes, and then combined the gene expression profile data and the GSVA algorithm to score functional pathway for each single cell. We found that there is no significant difference between dormant tumor cells and normal proliferating tumor cells in most signaling pathways, but there are significant differences in some signaling pathways. Therefore, we used a *t*-test to test the difference of GSVA scores in each signal pathway, and found that there are differences in the following signal pathways: COMPLEMENT, MYC_TARGETS_V1, MYC_TARGETS_V2 (Fig. 6). This showed that MYC targeting genes may be the key to tumor dormancy mechanism. Therefore, according to the GSVA model in the figure above, we took the genes that are significantly down-regulated in dormant cells and genes in MYC_TARGETS_V1 and MYC_TARGETS_V2 to intersect, three significantly down-regulated genes Ccna2, Mad2L1 and Plk1 related to MYC were obtained. We found that the expression of the oncogene Myc is slightly higher in the dormant tumor cells than in the proliferating tumor cells, but the Wilcoxon test p value is 0.2, indicating that the expression difference is not large, and this also shows that the Myc gene is indeed an important carcinogen gene, whether dormant or proliferating tumor cells require the expression of this gene. We found that the expression of the oncogene Myc is slightly higher in the dormant tumor cells than in the proliferating tumor cells, but the Wilcoxon test p value is 0.2, indicating that the expression difference is not large, and this also shows that the Myc gene is indeed an important carcinogen genes, whether dormant or proliferating tumor cells require the expression of this gene. After analyzing the targeted genes regulated by MYC, we found that Ccna2, Mad2L1 and Plk1 genes have obvious differential expression, indicating that dormant tumor cells are compared Proliferation of tumor cells does not affect Myc gene expression, but enters dormancy by interfering with Myc targeted genes (Fig. 7).

### Prognosis of PCa patients

**Fig. 8:**
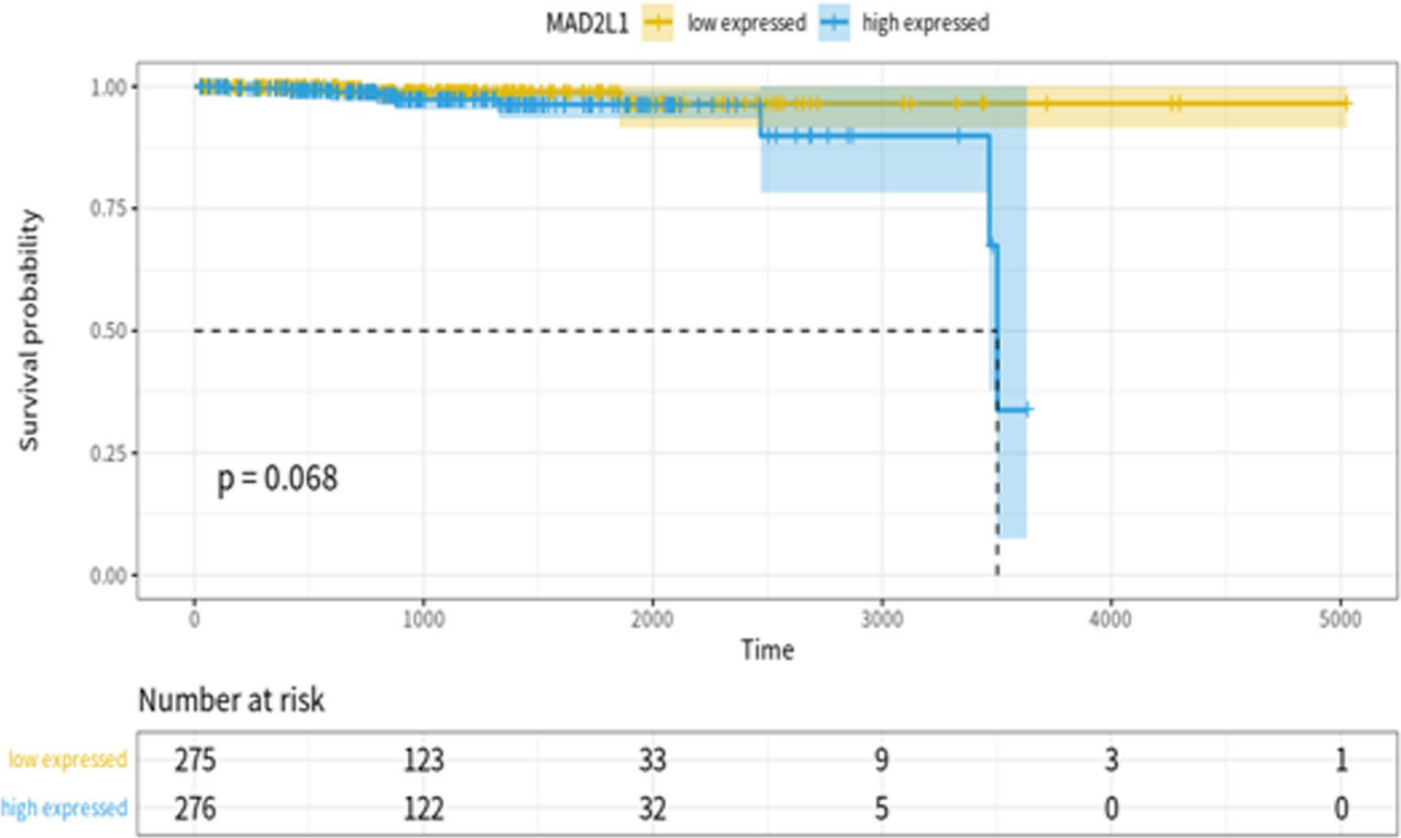
Correlation analysis between MAD2L1 gene and prognosis of patients with PCa in USCS Xena database

After the above analysis, we found that the MYC targeting gene MAD2L1 is potentially related to the tumor dormancy mechanism. Through the prognostic analysis of PRAD patients in TCGA, we hope to screen new biomarkers that can be used to predict the prognosis of patients. Among them, the MAD2L1 gene can be used to predict patient prognosis (log-rank test *P*-value = 0.068, *HR *[95% *CI*] = 3.184[0.8562–11.84]), especially after many years of disease, the prognosis of patients with high expression of MAD2L1 (expression value greater than the median) is significantly worse, indicating that if the MAD2L1 gene can be down-regulated to prevent tumor cells from proliferating in large numbers, it may help the prognosis of patient (Fig. 8).

## Discussion

The MYC gene family and its products are involved in the regulation of cell growth, differentiation and programmed death, and play an important role in the formation of most tumors [21, 22]. MYC is a transcription factor with a wide range of functions. It regulates cell proliferation and differentiation through a variety of mechanisms, such as the regulation and amplification of target genes, so the study of MYC gene and its related products may have positive significance for tumor detection and treatment [23–26].

MYC targeting gene MAD2L1, as an important part of the spindle assembly checkpoint (SAC), can be activated by the mitotic arrest deficient-like 1 (MAD1L1) and form a tetrameric complex with it, play an important role in starting the checkpoint signal. The abnormal expression of MAD2L1 will cause the weakening or disappearance of SAC function, making it impossible to monitor the abnormal combination of microtubules and kinetochores in tumor cells, causing abnormal separation of chromosomes, causing cell aneuploidy abnormalities and leads to the occurrence and development of tumors [27].

Xiang, et al. found that MAD2L1 was a significantly dysregulated hub gene in PCa compared with normal controls, indicating that MAD2L1 dysregulation may be closely associated with PCa malignant progression [28]. The abnormal expression of MAD2L1 is related to the progression of most tumors and the process of chemotherapy resistance; many types of human tumor cell chromosomal defects are related to the abnormal expression of MAD2L1. Morishita et al. reported the correlation between abnormal expression of MAD2L1 and locally advanced cervical cancer. By analyzing the relationship between the expression level of MAD2L1 and the prognosis of patients with cervical cancer, they found that the expression level of MAD2L1 can be used to predict the prognosis of locally advanced cervical cancer [29]. Zhang, et al. analyzed the expression of MAD2L1 in hepatocellular carcinoma and its clinicopathological significance and found that MAD2L1 was frequently overexpressed in hepatocellular carcinoma, including abnormal hepatocellular nodules and early lobular hepatocellular carcinoma, indicating that the overexpression of MAD2L1 plays a certain role in the occurrence and development of hepatocellular carcinoma. It may be an early event in the occurrence of hepatocellular carcinoma and can be used as a potential prognostic indicator [30]. In addition, abnormal expression of MAD2L1 has also been found in malignant tumors such as multiple myeloma, gastric cancer, and ovarian serous adenocarcinoma [31–33]. Therefore, MAD2L1 may also be an effective biomarker and prognostic molecule in PCa.

Tumor dormancy is one of the biological characteristics of malignant tumors. Tumor dormancy is also an important reason why tumors are difficult to cure, leading to metastasis and recurrence in patients. The results showed that Wnt5a in osteoblasts induces PCa cell dormancy by activating atypical ROR2/SIAH2 signals, resulting in typical Wnt/β-catenin signal inhibition [34]. Lee et al. injected the PCa cell C4-2B4 subcutaneously into mice and found that the PCa cell C4-2B4 was in a proliferating state under the skin. When the PCa cell C4-2B4 was dormant after bone metastasis, it was speculated that the bone microenvironment puts PCa cells C4-2B4 in a dormant state. Further studies have shown that occult diffuse tumor cells are induced to enter a dormant state through TGFBRII-p38MAPKpS249/pT252-RB signals [35].

In this study, we found for the first time that the MYC targeting gene MAD2L1 is associated with PCa bone metastasis tumor cell dormancy. By analyzing the differential genes of dormant tumor cells and proliferating tumor cells, a total of 378 differentially expressed genes were obtained, of which 293 genes were up-regulated, mainly related to immune response related genes; and 85 genes were down-regulated, mainly related to the cell cycle. Among them, the genes up-regulated by proliferating tumor cells are mainly related to the immune response, and the genes down-regulated by dormant tumor cells are mainly related to the cell cycle. The immune-related genes in dormant cells are not significantly up-regulated, indicating that dormant cells can escape the attack of the immune system. Down-regulation of cell cycle-related genes in dormant tumor cells is also consistent with the feature that dormant cells no longer have strong proliferation.

## Conclusion

This study reported for the first time that the MYC targeting gene MAD2L1 is potentially associated with the dormancy mechanism of PCa. Through prognostic analysis of PCa patients in TCGA, it was found that the MAD2L1 gene can be used to predict the prognosis of patients, especially after years of illness, the prognosis of patients with high expression of the MAD2L1 gene is significantly worse, indicating that if the MAD2L1 gene can be down-regulated to prevent tumor cells from proliferating in large numbers, it may help the prognosis of patient.

## Data Availability

The datasets used and analyzed during the current study are presented in the main body of this manuscript.
